# First Autochthonous Coinfected Anthrax in an Immunocompetent Patient

**DOI:** 10.1155/2015/325093

**Published:** 2015-09-15

**Authors:** Parvaneh Afshar, Mohammad Taghi Hedayati, Narges Aslani, Sadegh Khodavaisy, Farhang Babamahmoodi, Mohammad Reza Mahdavi, Somayeh Dolatabadi, Hamid Badali

**Affiliations:** ^1^Referral Laboratory, Mazandaran University of Medical Sciences, Sari, Iran; ^2^Invasive Fungi Research Center and Department of Medical Mycology and Parasitology, School of Medicine, Mazandaran University of Medical Sciences, Sari, Iran; ^3^Student Research Committee, Mazandaran University of Medical Sciences, Sari, Iran; ^4^Department of Medical Mycology and Parasitology, Kurdistan University of Medical Sciences, Sanandaj, Iran; ^5^Department of Medical Mycology and Parasitology, School of Public Health, Tehran University of Medical Sciences, Tehran, Iran; ^6^Antimicrobial Resistance Research Center, Department of Infectious Diseases, Mazandaran University of Medical Sciences, Sari, Iran; ^7^Mazandaran University of Medical Sciences, Sari, Iran; ^8^CBS-KNAW Fungal Biodiversity Centre, Utrecht, Netherlands; ^9^Cellular and Molecular Research Center, Sabzevar University of Medical Sciences, Sabzevar, Iran; ^10^Pharmaceutical Sciences Research Center and Department of Medical Mycology and Parasitology, School of Medicine, Mazandaran University of Medical Sciences, P.O. Box 48175-1665, Sari, Iran

## Abstract

Cutaneous anthrax has a mortality rate of 20% if no antibacterial treatment is applied. The clinical manifestations of cutaneous anthrax are obviously striking, but coinfection may produce atypical lesions and mask the clinical manifestations and proper laboratory diagnosis. Anthrax is known to be more common in the Middle East and Iran is one of the countries in which the zoonotic form of anthrax may still be encountered. We report a case of a 19-years-old male who used to apply Venetian ceruse on his skin. Venetian ceruse (also known as Spirits of Saturn) is an old cosmetic product used for skin whitening traditionally made from sheep's spinal cord. The patient referred to the Referral Laboratory, Mazandaran University of Medical Sciences, Sari, Iran, with atypical dermatosis, pronounced pain, and oedema of the affected tissue. It was confirmed by both conventional and molecular analysis that culture was a mixture of *Bacillus anthracis* and *Trichophyton interdigitale*. The patient was initially treated with ceftriaxone (1000 mg/day for two weeks), gentamicin (1.5–2 mg/kg/day), terbinafine (200 mg/week for one month), and 1% clotrimazole cream (5 weeks) two times per day which resulted in gradual improvement. No relapse could be detected after one-year follow-up. Anthrax infection might present a broader spectrum of symptoms than expected by clinicians. These unfamiliar characteristics may lead to delayed diagnosis, inadequate treatment, and higher mortality rate. Clinicians need to be aware of this issue in order to have successful management over this infection.

## 1. Introduction

Anthrax is an acute bacterial infection caused by Gram-positive, aerobic, spore-forming* Bacillus anthracis* which has a worldwide distribution [1]. Anthrax has been known by its three different clinical entities depending on the route of entry, that is, cutaneous, gastrointestinal, or inhalational anthrax [2]. The cutaneous type of anthrax is the most common form of the disease and initially occurs around the area of implantation. It can be caused due to direct contact with infected herbivorous animals and their flesh, bones, hides, hair, and excrement leading to localized, painless, central black eschar surrounded by nonpurulent oedema [3]. The clinical manifestations of cutaneous anthrax are obviously striking; thus, coinfection may produce atypical lesions misleading the clinicians and delaying the diagnosis of cutaneous anthrax. The head and hands are the most commonly affected parts of the body. It happens usually on exposed parts and it may give rise to local oedema that develops into a necrotic lesion followed by systemic infection.* Bacillus anthracis* is the causative agent of anthrax and its incidence was thought to be restricted to the Middle East and the Persian Gulf region where the zoonotic form of anthrax may still show a mortality rate of 20% if no antibacterial is applied [4, 5]. Although the older forms of anthrax have become extremely rare in Europe [6], new described cases are mostly among heroin users due to contaminated heroin distributed, for instance, in Denmark, France, Germany, and the United Kingdom [7], and can be considered as a novel clinical manifestation, that is, injectional anthrax [6, 8]. Anthrax is also a neglected zoonotic disease in African countries [9]. In this paper, we describe the first case of coinfection with anthrax and tinea barbae due to* B. anthracis *and* T. interdigitale *causing cutaneous infection in a 19-year-old male farmer and shepherd from Iran where anthrax is autochthonous.

## 2. Case Report

**Figure 1:**
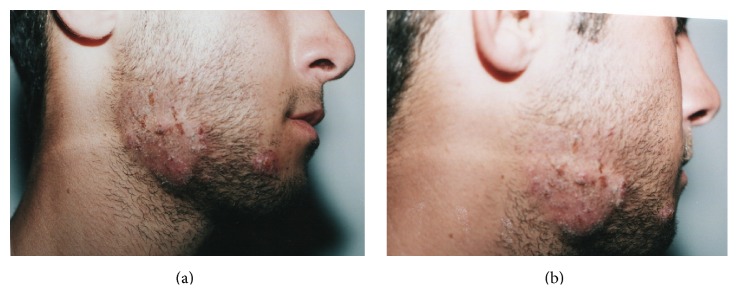
Lesions characteristic for cutaneous anthrax and dermatophytosis due to* Bacillus anthracis* and* Trichophyton interdigitale*.

**Figure 2:**
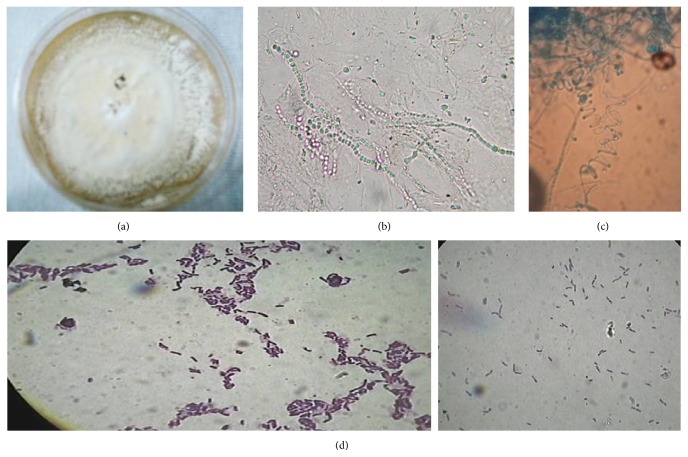
(a) Culture on Sabouraud dextrose agar (SDA, Difco) produced flat, white-to-cream colonies with a powdery-to-granular surface and yellow-brown colour in reverse; (b) hyaline, smooth-walled, and spherical-to-subspherical microconidia; (c) spiral hyphae; (d) nonbranching Gram-positive bacilli.

A 19-year-old healthy male, farmer and shepherd, native and resident in Northwest of Iran, was admitted to the Department of Infectious Diseases of Mazandaran University of Medical Sciences, Sari, Iran, and presented with atypical dermatosis and fever, with the right side of his face swollen. Though the patient had much contact with sheep and slaughtering the sheep, he could not remember any history of trauma or puncture at the site of the lesion. The symptoms started two weeks prior to the emergence of the lesion, initially as a painful vesicle after having contact with Venetian ceruse (Spirits of Saturn) which is traditionally made from sheep's spinal cord. Initially, the lesions were small, asymmetric, swollen, and painless but the size has increased gradually and itchy nodule developed afterwards as pruritic scaly erythema with small dark dots at the surface and serous discharge by firm pressure was noticed (Figures 1(a) and 1(b)). Due to the atypical clinical signs and occupational history of the patient, the initial suspicion was soft tissue infection or deep venous thrombosis. Nevertheless, no significant regional lymphadenopathy was noticed. No underling diseases were found and he was considered as an immunocompetent person. The blood count, blood chemistry, and renal functions were normal. Serological test for human immunodeficiency virus (HIV) and hepatitis C antibodies was negative. Ceftriaxone (800 mg/day) and gentamicin (1.5–2 mg/kg/day) treatments were started for suspected bacterial infections; however, fungal infection and other disorders were still considered. The skin scarping and serous discharges obtained from the lesions on his face were used for mycological and bacteriological investigations at the reference laboratory center. Gram staining of the specimens revealed nonbranching Gram-positive bacilli growing in chains and* Bacillus anthracis *was confirmed in aerobic blood culture (Becton Dickinson, Heidelberg, Germany). On subcultures, the bacilli were sticky, rough, white-grey, nonhaemolytic, and lipase negative on egg yolk agar (HiMedia, India). Phase-contrast microscopy showed large nonmotile bacilli and pearl string test was positive. Catalase test was positive. Glucose, fructose, maltose, and sucrose tests were positive with production of acid. These findings confirmed that the organism was correctly identified as* Bacillus anthracis*. Antibacterial susceptibility testing was performed using disk diffusion test. The isolate was susceptible to ceftriaxone, meropenem, penicillin, clindamycin, vancomycin, tetracycline, ciprofloxacin, and moxifloxacin but resistant to rifampin. The MIC result showed that ceftriaxone and penicillin have the highest efficiency. In addition, direct examination (KOH 10%) revealed indistinct, hyaline septate fungal hyphae. Subsequently, the remaining samples were inoculated on Sabouraud dextrose agar (SDA, Difco) with and without chloramphenicol (50 mg/L) and incubated at 30°C in the dark for two weeks. Cultures became positive with a fungus and routine morphological identification revealed a species of* Trichophyton* as causative agent. Stock cultures were maintained on slants of 2% malt extract agar (MEA, Difco) at 24°C, and a voucher strain was deposited into the reference laboratory culture collection of Mazandaran University of Medical Sciences under accession number LCCM10. Slides from 3- to 4-week-old cultures were prepared in lactophenol cotton blue and examined with a Nikon Eclipse 80i microscope equipped with a Nikon digital sight DS-Fi1 camera. Colonies grew fairly rapidly and were flat, white to cream in colour, with a powdery-to-granular surface and yellow-brown color in reverse (Figure 2(a)). Numerous single-celled microconidia were formed, and hyaline, smooth-walled, and spherical-to-subspherical microconidia, occasionally clavate to pyriform, were observed. Spherical chlamydoconidia, spiral hyphae, and smooth, thin-walled, clavate shaped, multicelled macroconidia were present which indicated the identification of* T. interdigitale* (Figures 2(b) and 2(c)). DNA was extracted using an Ultra Clean Microbial DNA Isolation Kit (Mobio, Carlsbad, CA, USA) according to the manufacturer's instructions [10]. Molecular identification was performed based on ITS rDNA region [11]. ITS rDNA was amplified using primers V9G (5′-TTACGTCCCTGCCCTTTGTA-3′) and LS266 (5′-GCATTCCCAAACAACTCGACTC-3′) and sequenced with the internal primers ITS1 (5′-TCCGTAGGTGAACCTGCGG-3′) and ITS4 (5′-TCCTCCGCTTATTGATATGC-3′). The final sequence was blasted and compared with GenBank database and was identified as* T. interdigitale* showing 99.5% sequence identity with the ex-type of the species. After final confirmation about the presence of both Gram-positive* B. anthracis* and fungus* T. interdigitale*, the ceftriaxone dose was increased to 1000 mg/day for two weeks, terbinafine to 200 mg/week for one month, and 1% clotrimazole cream two times for 5 weeks, which resulted in gradual improvement. However, surgical intervention was not considered due to the absence of any abscesses. During the following month, the condition of the patient slightly improved, no relapse in one-year follow-up was observed, and the patient was considered to have been successfully cured. The presentation of this case was approved by the ethics committee of Mazandaran University of Medical Sciences, and written informed consent was obtained from the patient for publication of this report.

## 3. Discussion

Anthrax is a zoonotic infection with a worldwide distribution, transmitted from animal to animal or to human. Nowadays, cases of anthrax are extremely rare due to the vaccination of high-risk people and animals. During 1989–1995, there were 1626 suspected cases of anthrax in cattle reported in Zambia and of these 51 were confirmed. Challenges to control anthrax are complex and comprise sociopolitical, economical, environmental, and cultural factors [9]. In Zimbabwe, anthrax outbreaks are reported annually [12]. An anthrax epidemic in Zimbabwe caused more than 6000 cases and about 100 deaths. In a study performed by Chikerema et al., in rural communities of Zimbabwe, 41.4% of the farmers were from high-anthrax-risk districts, whereas 28.5% and 30.1% were from medium- and low-risk districts, respectively [13]. Skin is the most frequent route of entry for* Bacillus anthracis* spores into the body. Anthrax is generally an occupational hazard and people working with infected animal or contaminated animal products (especially goat hair) are at risk of infection [14, 15]. Nowadays, the incidence of cutaneous anthrax is rare, sporadic cases are easily overlooked, and the diagnosis often is not considered [16]. It is unlikely for a physician in developed countries to come across an anthrax patient; therefore, the diagnosis may be difficult. This disease should be considered in any patient who shows painless ulcers associated with vesicles and oedema and history of direct contact with animal's products [17], but coinfection or secondary infection may mask the lesion and appear like an ordinary infected ulcer as presented in the current case. Outbreaks of anthrax have been reported in animals after ingestion of feedstuffs containing meat or bone meal-based concentrates made from carcasses contaminated with anthrax spores [18]. In our case, the disease might have been transmitted by direct contact with infected sheep or sheep's products like Venetian ceruse which is made from sheep's spinal cord and this patient was regularly using it for skin cleaning. The patient did not recall any trauma or injuries, but it is possible that spores might have invaded the dermis through microscopic epidermal defects. Babamahmoodi et al. [19] reported three rare cases of anthrax (gastrointestinal, oropharyngeal, and meningitis) from the same source in northern Iran (Mazandaran province) which was coming from a single family after consuming half-cooked meat of the contaminated sheep. In addition, Erkek et al. [20] presented an unusual extensive case of cutaneous anthrax in a patient with type II diabetes mellitus; although cutaneous anthrax is usually self-limiting, complications may arise in untreated cases. Durić et al. [21] reported three probable cases of cutaneous anthrax in the eastern part of the autonomous province of Vojvodina, Serbia. All cases were involved in slaughtering of a heifer that was suspected to have anthrax. In the same village, anthrax was confirmed in other animals. Recently, several heroin-associated anthrax cases have been reported from Europe which can become a new continuous source of injectional anthrax across Europe [6, 8, 22]. Epidemiological evidence still implicates Venetian ceruse (skin whitener) as one of the main sources of spore transmission in long term application as was seen in our case reported here. Venetian ceruse may be a continuous source of anthrax in the Middle East leading to both outbreaks and sporadic cases. It is assumed that the contamination with anthrax spores occurs during either direct contact with infected sheep or by using Venetian ceruse.* Bacillus anthracis*, the causative agent of anthrax, is susceptible to antibiotics and treatment is effective in the early stages; however, treatment of other agents due to coinfections is crucially important, as in the case reported here.* T. interdigitale* which is a filamentous fungus should be treated with antifungal agents. Therefore, early diagnosis of the cocutaneous anthrax is important for effective treatment. Clinicians should recognize that cutaneous anthrax infection might present with a broad spectrum of symptoms; therefore, these unfamiliar characteristics have led to delays in diagnosis, inadequate treatment, and a high mortality rate. Therefore, clinician's awareness is crucially important for early and successful management of infections.
